# Supplementary material for: Spatial control over catalyst positioning on biodegradable polymeric nanomotors
Source: Nat Commun. 2019 Nov 22;10:5308. doi: 10.1038/s41467-019-13288-x (PMC6876569; doi:10.1038/s41467-019-13288-x)
Supplement: Supplementary file 1 — Supplementary Information [file 41467_2019_13288_MOESM1_ESM.pdf]

SUPPLEMENTARY INFORMATION

Spatial control over catalyst positioning on biodegradable polymeric nanomotors

B. Jelle Toebes, F. Cao and Daniela A. Wilson*

Supplementary Methods

All compounds were used as received. For synthesis of the three PEG-PDLLA copolymers, methoxy-PEG_{22}-OH (1 kDa) was purchased from Creative PEG Works and methoxy-PEG_{44}-OH (2 kDa) and N_3-PEG_{67}-OH (3 kDa) were purchased from Rapp Polymere. D,L-Lactide and the tetrahydrofuran (THF) were purchased from Acros Organics. The ultra-pure Milli-Q water was obtained via Labconco Water Pro PS purification system (18.2 ME). Dialysis membranes of MWCO 12-14000 Dalton Spectra/Por were used to remove the organic solvent. Sodium chloride was purchased from Merck. The DBCO-Sulfo-NHS was acquired from Click Chemistry Tools. The Alexa Fluor 647-NHS ester and the Pierce immobilized TCEP resin (4% crosslinked on silica beads, effective functional TCEP concentration > 8 mM) were supplied by Thermo Scientific. Dioxane was purchased from Biosolve Chimie. The Amicon Ultrafree centrifugal filters were purchased from Merck-Millipore. Materials for the SDS-PAGE were bought from Bio-Rad, including the Mini-Protean TGX Stain-Free Gels (4-20%, 12 well) and the Precision Plus Unstained marker. All other chemicals, including the NHS functionalized gold particles (20 nm) conjugation kit and the Amplex red enzyme activity assay kit, were supplied by Sigma-Aldrich.

Nuclear Magnetic Resonance (NMR) was measured at 298 K on a Bruker 400 MHz Avance III HD nanobay spectrometer equipped with a 9.4 T Ascend magnet (400 MHz) and BBFO probe. Chemical shifts are given in parts per million (ppm) with respect to tetramethylsilane (TMS, δ 0.00 ppm) as internal standard for ^{1}H NMR. ^{1}H spectra were acquired using 48 scans and a relaxation delay of 9 seconds.

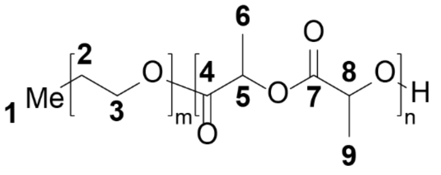

Poly(ethylene glycol)$_m$-b-poly(D,L-lactide)$_n$: **^{1}H NMR** ($CDCl_3$, 400MHz) δ 5.19 (m, 2n H, 5-C*H* + 8-C*H*), 3.64 (s, 4m H, 2-C*H*$_2$ + 3-C*H*$_2$), 3.38 (s, 3 H, 1-C*H*$_3$), 1.66-1.50 (m, 6n H, 6-C*H*$_3$ + 9-C*H*$_3$). **^{13}C NMR** ($CDCl_3$, 101MHz)**:** δ 169.58 (4-C + 7-C), 71.08 (2-C + 3-C), 68.61 (5-C + 8-C), 58.97 (1-C), 16.31 (6-C + 9-C).

Dynamic Light Scattering (DLS) measurements were performed on a Malvern Instruments Zetasizer (ZEN 1600), using Zetasizer Software (Malvern Instruments) for analysis of the data. Samples were loaded in Malvern disposable capillary cells. The average of three size measurements with 10 scans of 10 seconds was taken.

Cryogenic Transmission Electron Microscopy (cryo-TEM) pictures were taken on a JEOL TEM 2100 microscope (JEOL Japan). Analysis and processing of the data was performed using Fiji (a free program developed by NIH and available at https://fiji.sc/).
Protocol: EM Science TEM grids were glow discharged with a 208 carbon coater (Cressington). On each grid 3 µL of sample was added, blotted and immediately vitrified through freeze plunging into liquid ethane at 100% humidity using an automatic vitrification robot, FEI Vitrobot™ Mark IV (blot time 1 s, blot force 3). Samples were loaded in a 914 High tilt cryoholder (Gatan, Munich, Germany) and inserted into a JEOL Transmission Electron Microscope 2100 (Japan) at 200 kV. Images were taken with a 4096 x 4096 pixel CCD camera (Gatan). The average dimensions and membrane thickness of each sample were obtained from different regions (images) and analyzed with plot profile tools of Fiji.

Confocal imaging was done on a Leica (Wetzlar, Germany) SP8 confocal microscope equipped with a HC PL APO CS2 40x/1.10 WATER immersion objective. The Detector used was HyD (658nm - 783nm) Standard mode. Bidirectional scan direction X and a scan speed of 600 Hz was used. The samples were loaded in an Ibidi glass bottom 8 well µ-slide.

UV-VIS Absorbance was recorded on JASCO V-630 UV-Vis spectrophotometer using a 3.5 mL quartz cuvette with a path length of 1.00 cm.

Fluorescence Spectra were recorded by JASCO FP-8300ST Spectrofluorometer, using excitation at 490 nm and emission from 500-700 nm, respectively.

Nanoparticle Tracking Analysis (NTA) was performed on a Nanosight LM10 at 20x magnification. This technique combines laser light scattering with a CCD camera (30 fps) to track individual particles between 30-1000 nm size in real time. It uses the Stokes-Einstein equation ($D = \frac{T*kB}{3\pi\eta d}$, where D is the particle diffusion coefficient, kB the Boltzmann constant, η the viscosity, T the temperature and d the hydrodynamic diameter) to correlate the tracking coordinates obtained from the displacement of the particles with their size (5). In this experiment we analyzed the movement of stomatocytes filled with catalase or catalase and glucose oxidase with addition of three concentrations of fuel.

Protocol: 10 µl of nanomotor solution was diluted in 1 mL PBS buffer (0.05 M, pH 7), containing different amounts of fuel (Video S1 and 2). After injection in the cell, videos of 60 seconds were taken and processed by the NTA2.2 software. By analyzing the video, x and y coordinates of each particle were determined as a function of time intervals. Mean square displacements obtained for 100 frames by averaging over at least 100 particles per sample were plotted versus the time intervals (Figure S3).

Complete reduction of the azide handles:

To a suspension of azide functionalized stomatocyte vesicles (200 µL of 5.0 mg/mL polymersomes), 100 µL of 1.00 mg/mL tris(2-carboxyethyl)phosphine (TCEP) (160 eq. relative to max. available azide-groups) was added. Unreacted TCEP was removed by spin filtration and several washing steps with 0.22 µm spin filters for 10 min at 2655 rcf.

Enzyme activity assay:

The enzyme activity of catalase was determined by an Amplex red hydrogen peroxide assay. In this assay, the Amplex Red reagent competes for hydrogen peroxide to produce the fluorescent product, resorufin. A calibration curve with different concentrations of catalase was made, starting from 0.1 – 4 U. The formed fluorescent resorufin was measured by a Tecan Spark M10 plate reader (excitation at 550 nm and Emission at 590 nm). Catalase samples with various ratios of DBCO coupled to the enzyme were tested and compared to the calibration curve for their activity.

Supplementary Tables

Supplementary Table 1: *Overview of PEG-PDLLA block copolymer compositions*

Polymer	Polymer composition (NMR)	PDI (GPC)
PEG_{22}-$PDLLA_{90}$	PEG_{22}-$PDLLA_{91}$	1.08
PEG_{44}-$PDLLA_{90}$	PEG_{44}-$PDLLA_{98}$	1.05
N_3-PEG_{67}-$PDLLA_{75}$	N_3-PEG_{67}-$PDLLA_{79}$	1.10

Supplementary Figures

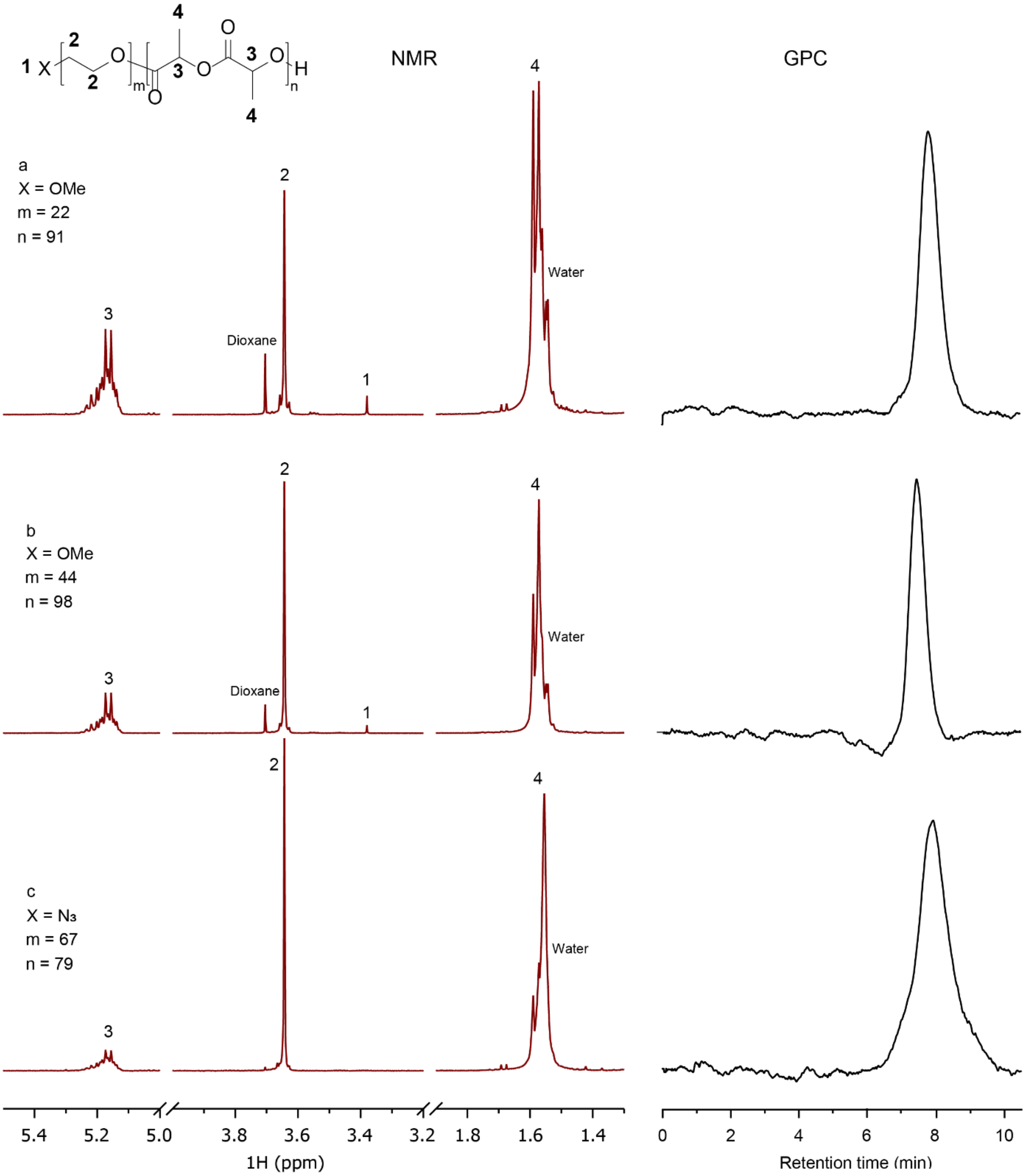

Supplementary Figure 1: Characterization of PEG-PDLLA polymers. NMR spectrum and GPC chromatogram of **a,** PEG_{22}-$PDLLA_{90}$ **b,** PEG_{44}-$PDLLA_{90}$ **c,** N_3-PEG_{67}-$PDLLA_{75}$. Compositions are calculated by integrating peaks 2 and 3 relatively to peak 1.

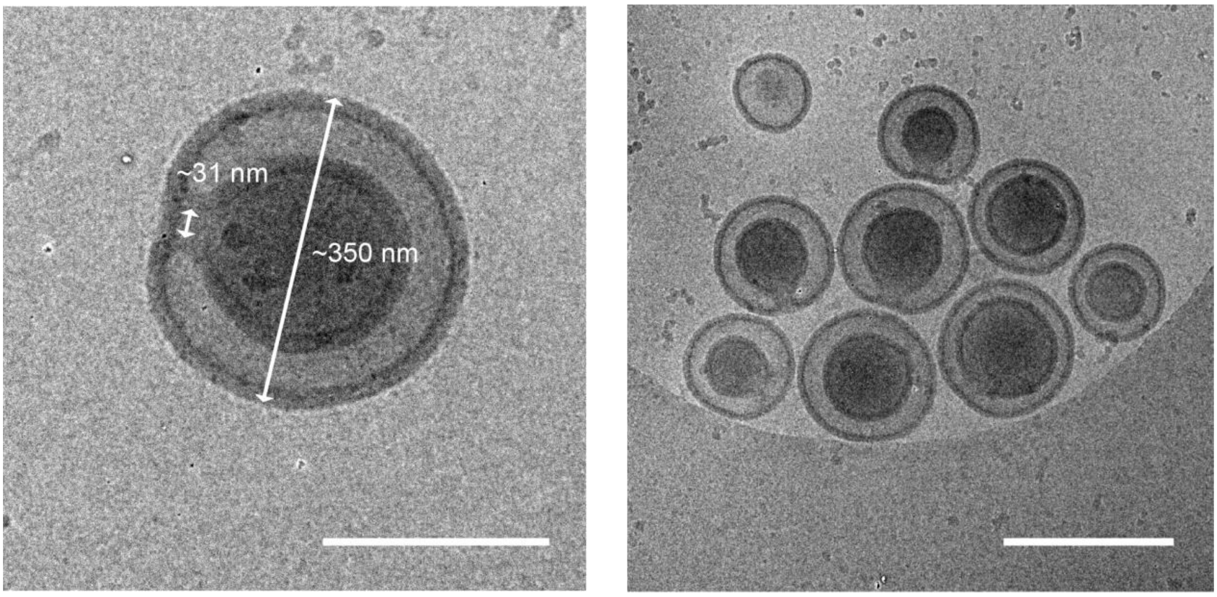

Supplementary Figure 2: Cryo-TEM pictures of nanomotors after functionalization with enzymes. The morphology of the stomatocyte motors are not affected by the functionalization processes. Diameter of the PEG-PDLLA stomatocyte and the size of the opening are measured with Fiji and averaged over 30 particles. Scale bars, 250 and 500 nm, respectively.

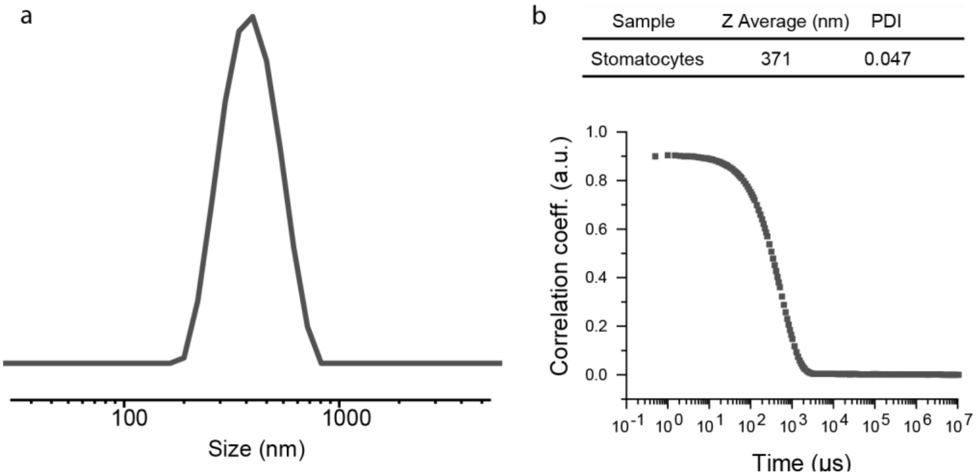

Supplementary Figure 3: Size overview of PEG-PDLLA stomatocytes by DLS. a, Size distribution by intensity. **b,** Average size and particle-size distribution index and correlation coefficient for quality analysis.

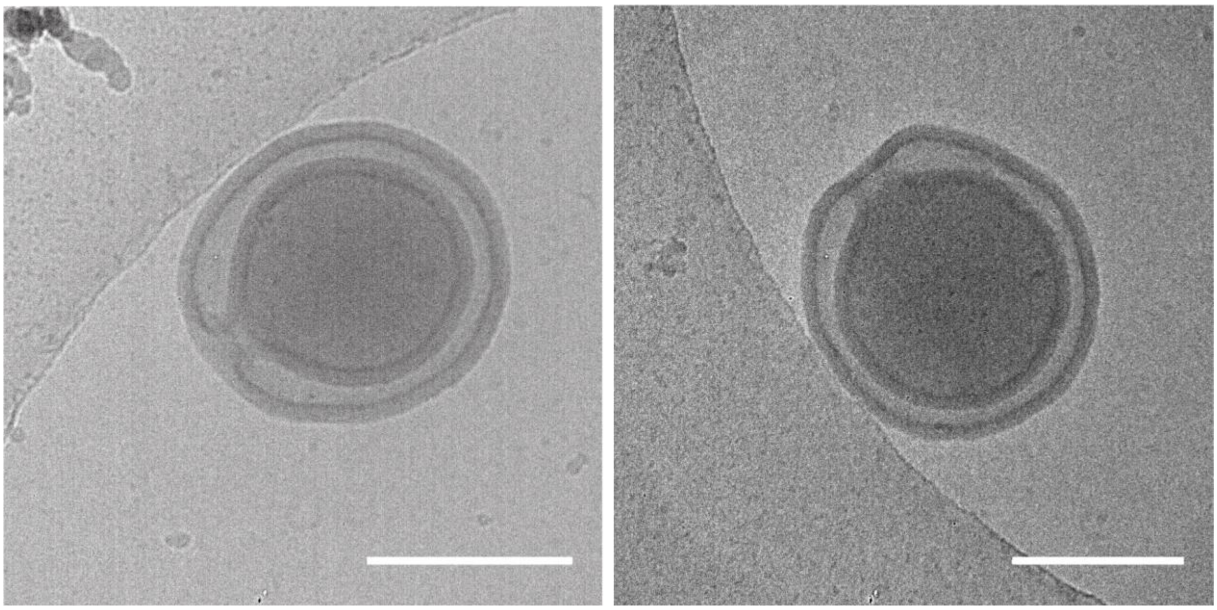

Supplementary Figure 4: Cryo-TEM picture of azide functionalized stomatocytes together with NHS functionalized gold nanoparticles as negative control. PEG-PDLLA stomatocytes without reduction after reaction with NHS functionalized gold nanoparticles, showing no binding as expected. Scale bars, 250 nm.

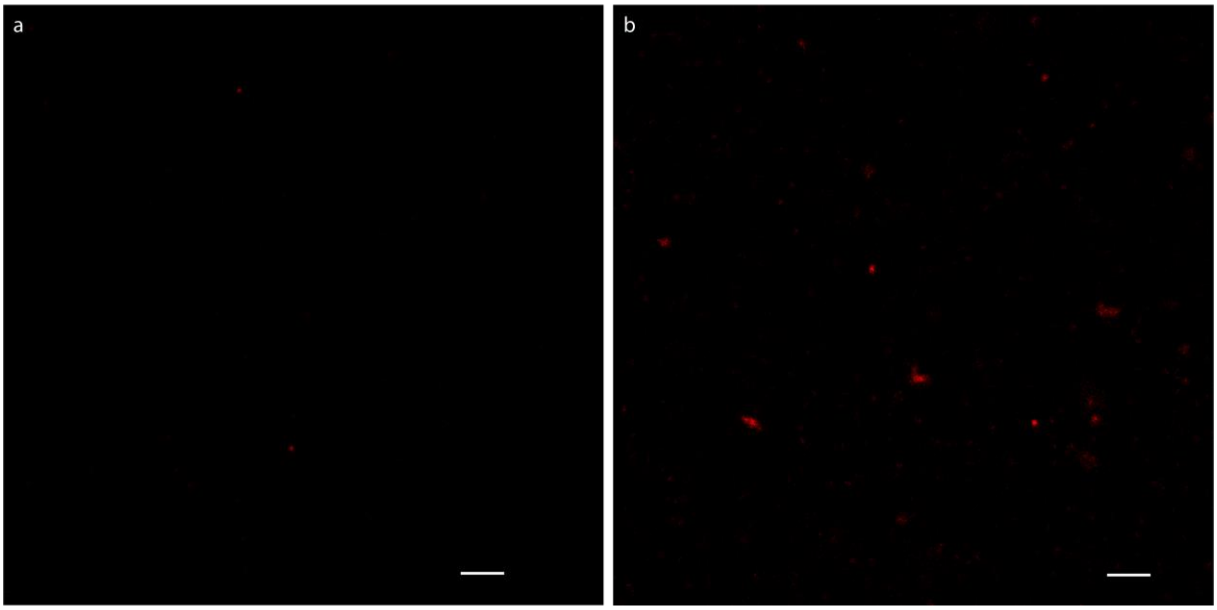

Supplementary Figure 5: Confocal pictures showing fluorescence signal of Alexa Fluor 568-NHS dye after reaction with **a,** stomatocytes without reduction, showing no binding as expected and **b,** stomatocytes reduced with TCEP beads, showing binding of the dye. Scale bars, 1 µm.

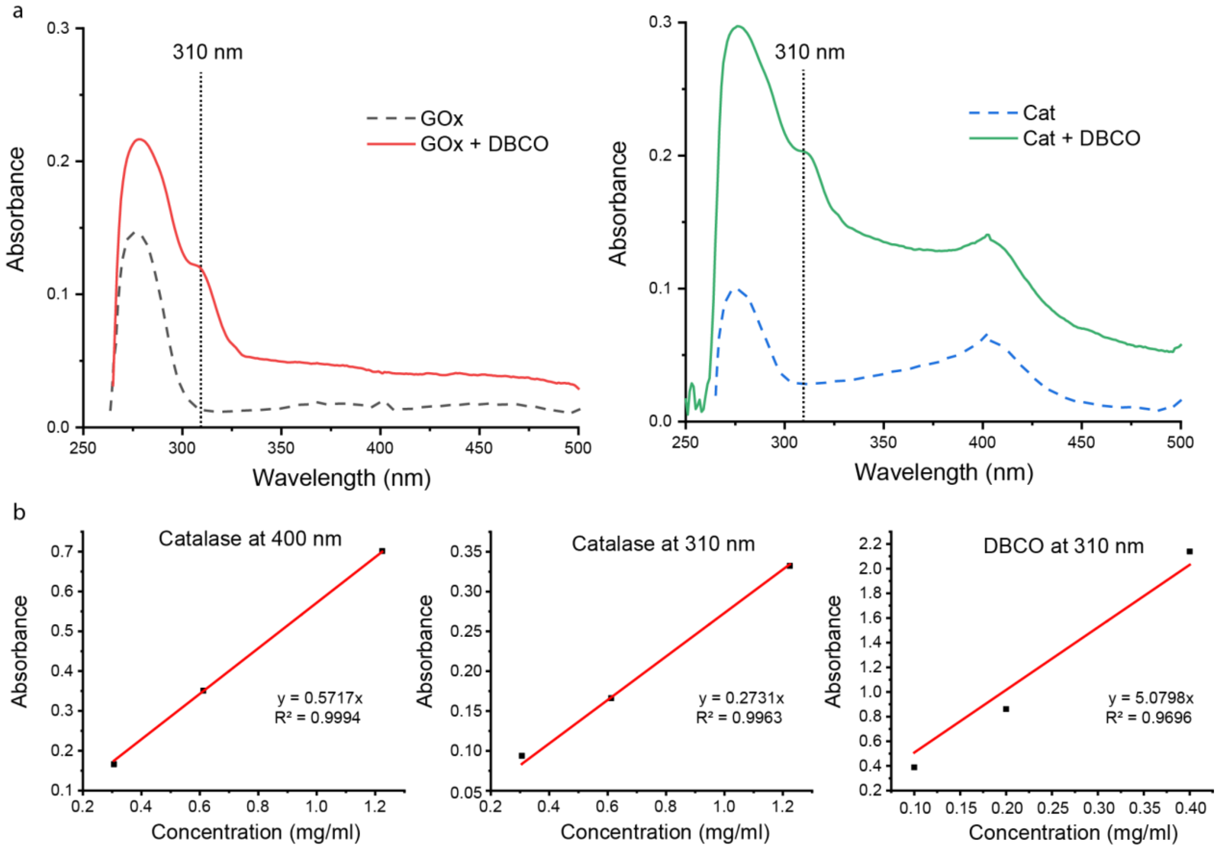

Supplementary Figure 6: UV-VIS spectra of **a,** glucose oxidase (GOx) and catalase (Cat) before and after coupling to DBCO-NHS. Absorbance peaks at 310 nm specific for the DBCO molecule show successful coupling. **b,** Absorbances of different concentrations of catalase and DBCO-NHS at various wavelengths, used to calculate the ratio of linker molecules per enzyme. The UV-VIS measurements show 11.8 linker molecules for each enzyme.

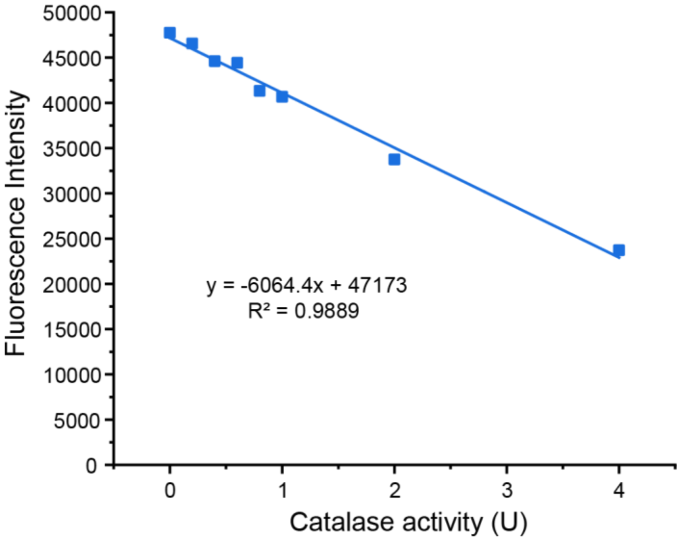

Supplementary Figure 7: calibration curve from the amplex red assay. From the calibration curve the activity of the DBCO coupled enzymes were estimated, showing 98.9 and 96.2% activity for 10:1 and 30:1 linkers per catalase, respectively.

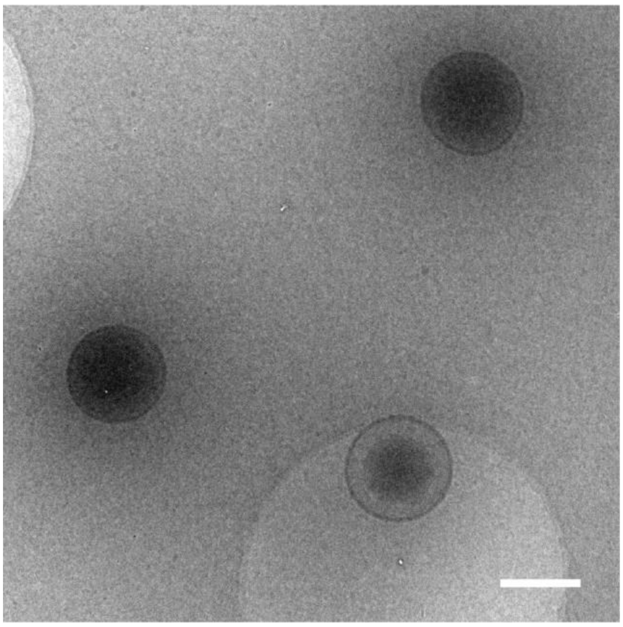

Supplementary Figure 8: Cryo-TEM picture of 5 wt% azide functionalized polymersomes.

The spherical morphology was obtained after dialysis against water instead of salt. Scale bar, 250 nm.

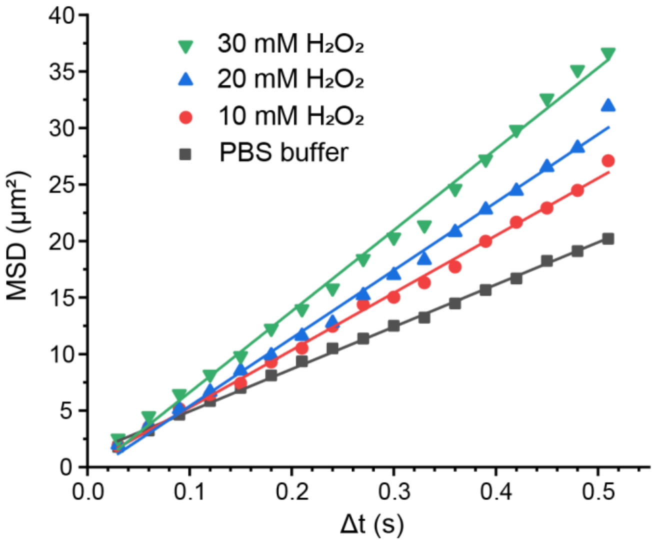

Supplementary Figure 9: Mean Squared Displacement (MSD) values of spherical polymersomes with catalase bound, showing enhanced diffusion in presence of fuel, 30 mM H_2O_2 (green triangles pointing down), 20 mM H_2O_2 (blue triangles pointing down), 10 mM H_2O_2 (red circles), PBS buffer (grey squares).
