# Supplementary material for: Spatial control over catalyst positioning on biodegradable polymeric nanomotors
Source: Nat Commun. 2019 Nov 22;10:5308. doi: 10.1038/s41467-019-13288-x (PMC6876569; doi:10.1038/s41467-019-13288-x)
Supplement: Supplementary file 3 — Description of Additional Supplementary Files [file 41467_2019_13288_MOESM3_ESM.pdf]

Description of Additional Supplementary Files

File Name: Supplementary Movie 1
Description: Nanosight video tracking stomatocyte nanomotors with catalase and glucose oxidase in PBS buffer without fuel.

File Name: Supplementary Movie 2
Description: Nanosight video tracking stomatocyte nanomotors with catalase and glucose oxidase in PBS buffer with 10 mM glucose.

File Name: Supplementary Movie 2
Description: Nanosight video tracking stomatocyte nanomotors with catalase in PBS buffer with 10 mM H2O2.
